# A Pilot Study of Non-invasive Sacral Nerve Stimulation in Treatment of Constipation in Childhood and Adolescence

**DOI:** 10.3389/fped.2020.00169

**Published:** 2020-04-16

**Authors:** Manuel Besendörfer, Martin Kohl, Vera Schellerer, Roman Carbon, Sonja Diez

**Affiliations:** Friedrich-Alexander-Universität (FAU) Erlangen-Nürnberg, Department of Surgery, Section Pediatric Surgery, University Hospital Erlangen, Erlangen, Germany

**Keywords:** pediatric surgery, electrostimulation, non-invasive sacral nerve stimulation, slow-transit constipation, Hirschsprung's disease

## Abstract

**Background/Aims:** Constipation shows both, a high prevalence and a significant impact. However, it is often perceived as minor and treatment choices are limited. The neuromodulation approach is a valuable option to be considered. This study assesses the use of non-invasive sacral nerve stimulation to reduce constipation in children.

**Methods:** Between February 2013 and May 2015, pediatric patients with chronic constipation were treated with this non-invasive neuromodulation procedure, adapted from classical sacral nerve stimulation. A stimulation device attached to adhesive electrodes on the lower abdomen and back generated an electrical field with a stable frequency of 15 Hz via variable stimulation intensity (1–10 V). The effect of therapy was evaluated in routine check-ups and by specialized questionnaires.

**Results:** The study assessed non-invasive sacral nerve stimulation in 17 patients (9 boys, 8 girls, mean age 6.5 years). They underwent stimulation with 6–9 V for a mean of 11 h per day (range 0.5–24 h) over a mean of 12.7 weeks. Improvement of constipation was achieved in more than half of the patients (12/17) and sustained in almost half of these patients (5/12). Complications were minor (skin irritation, electrode dislocation).

**Conclusions:** Non-invasive sacral nerve stimulation appears to be effective in achieving improvement in pediatric patients with chronic constipation. As an additional external neuromodulation concept, this stimulation may represent a relevant addition to currently available therapeutic options. Further studies are needed to confirm these results.

## Introduction

The high prevalence of constipation and an increasing understanding of gastrointestinal function have encouraged interest in new therapeutic approaches (1–3). Although obesity, with its effects, is perceived as a public health problem, constipation is sometimes trivialized. Affected patients have a high level of psychological distress (4), leading to a reduced quality of life (5, 6), which both is resulting in increasing health-related costs (7).

Constipation is a heterogeneous complex of symptoms (8). Incidences are thus strongly dependent on selection criteria and are broadly distributed. Data on incidences in children are sparse. Prevalence rates in adults are observed at 24.2% by an Italian study using the Rome III criteria, whereas subjective assessment ranks at 34.1% (9). The Rome III Classification System (10) defines criteria for chronic constipation as three or fewer defecations per week, difficult stool straining, a sensation of anorectal obstruction or incomplete evacuation, and/or manual maneuvers to aid defecation in at least 25% of occasions. Furthermore, loose stools must rarely be present without laxatives, and irritable bowel syndrome is excluded. With at least two of these symptoms present for at least 3 months, chronic constipation is diagnosed (11).

Whereas, therapeutic options are limited, sacral neuromodulation (SNS) is a promising option in adult treatment. SNS can affect multiple physiological functions of the pelvis and lower abdomen, and it supports propulsive peristalsis of the intestine, which is of special interest in slow-transit constipation (STC) (12, 13). Additionally, SNS was reported to reduce the need for anterograde colonic lavage in chronically constipated patients who used MACE (Malone antegrade continence enema) (14).

In pediatric patients, a child-friendly application of electrostimulation therapy is desirable. However, the large size of the devices is incompatible and the implanted electrodes are likely to dislodge due to child's activity and body growth. Thus, SNS has rarely been used in pediatric patients (15–17).

However, non-invasive neuromodulation for pediatric patients with chronic constipation has been presented by multiple authors with promising results so far (18–24). Nevertheless, there are important differences in stimulation variables and outcome of patients. This study explored the impact of non-invasive sacral nerve stimulation, a transabdominal neuromodulatory approach via electrical stimulation in treatment of pediatric patients by analyzing improvement in symptoms. The patients presented with chronic constipation of heterogeneous origin.

## Patients and Methods

Between February 2013 and May 2015, more than 70 patients with chronic constipation (>3 months) were treated in our pediatric surgery department. All patients run through a diagnostic course, including medical and behavioral treatment. Diagnosis was made in case of both, clinical signs of defecation disorder and pathological results in imagine diagnostics (contrast-based X-ray). Medical treatment had already been started by pediatricians in every case. After therapy with a plurality of options and their combinations, symptoms persisted. We defined failure of conservative medical treatment in cases of chronic constipation >1 year as inclusion criteria and proposed stimulatory therapy to all of these complex patients, irrespective of underlying diagnoses or secondary diagnoses such as encopresis or enuresis. Accordingly, 23 patients were entered into this pilot study. Clinical patient data were recorded with emphasis on accompanying illness, previous operations, genesis of the disease, and concomitant medical treatment. The study was conducted according to the Declaration of Helsinki and was approved by the local ethics committee. Consent was obtained from all included patients.

### Clinical Management of Non-invasive Sacral Nerve Stimulation

The non-invasive sacral nerve stimulation consists of a stimulator [Model 3625 Interstim®, Medtronic GmbH, 40670 Meerbusch (www.medtronic.com) (25)] connected via cable to a pair of body-adhesive electrodes [Stimex®-electrodes, Pierenkemper GmbH, 35630 Ehringshausen (pierenkemper.eu)]. The stimulator allows adjustments of voltage, pulse width, and frequency.

**Figure 1:**
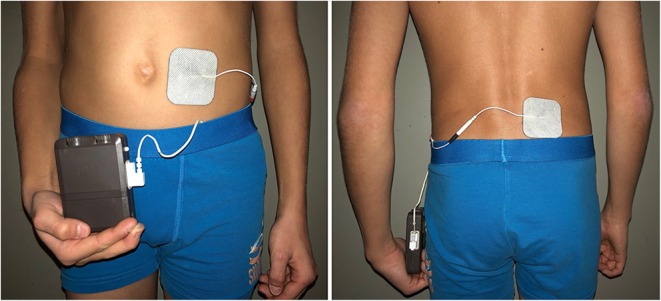
Placement of the adhesive electrodes: the ventral electrode is set paraumbilically on the left side, the dorsal one paravertebrally on the right side.

Two adhesive electrodes were applied. The first was placed paraumbilically on the left lower abdomen, the dorsal electrode paravertebrally on the right side near the lumbar spine (Figure 1). This diagonal positioning enables a maximum electrical field.

Unipolar stimulation was applied equally in every patient: pulse width was set at 210 μs, frequency at 15 Hz. Stimulation intensity could be adjusted by patients at any time between 1 and 10 V; however, it was recommended to set the voltage between 6 and 9 V. Daily duration of stimulation was restricted to 6–8 h in the trial phase. After proven tolerability for 7 days, the duration of stimulation could be extended up to 24 h per day based on patients' well-being and compliance.

The patients and their parents were advised to apply the stimulation for as long as possible (per 24 h), although it was acknowledged that the pacemaker should not restrict normal daily activity. Additionally, the patients/parents were instructed to switch off the pacemaker during defecation and micturition. The stimulation intensity was adapted to symptomatic changes to maximize the clinical benefit. Patients' parents were instructed to observe and document their child's behavior. If restlessness or discomfort was noted, the intensity of stimulation was reduced, and all individual adjustments were documented in a provided checklist. A questionnaire was used to address issues related to the application and effect of stimulation.

Patients were contacted weekly to monitor progress and address side effects immediately. Determined questions on defecation frequency, stool consistency, soiling and quality of life were assessed routinely in a structured manner by the attending physician (see Supplementary File 1: non-invasive sacral nerve stimulation - evaluation form). In cases of insufficient efficacy, the position of the electrodes was changed after 2 weeks of ineffective therapy (new position: paraumbilically on the right lower abdomen and on the left side near the lumbar spine). The first follow-up appointment was conducted at 4 weeks and evaluated the clinical benefit and individual adjustments to stimulation (intensity, duration of stimulation). Therapeutic benefit was defined in cases of elevated stool frequency, improved stool consistency, and less abdominal pain, which was described by the parents and confirmed in physician's appointments. Improvement of quality of life was evaluated, focusing on abdominal pain, patient's activity and patient's autonomy concerning defecation. Change of previous medication/defecation maneuvers, e.g., laxatives or rectal manipulations, was not recommended or conducted during this initial test phase. Effectiveness of stimulation therapy was defined in case of increased defecation frequency, reduced abdominal pain and/or reduced soiling.

After this therapeutic period of 4 weeks, stimulation was paused for 1 week to ascertain relapse or preservation of symptom improvement. If symptoms had improved sufficiently, stimulation was continued afterwards; otherwise, it was terminated. If therapy had shown only a slight subjective improvement (for example improved agility, less abdominal pain), the duration and level of stimulation were intensified as tolerated after this 1 week of therapeutic pause. If no improvement resulted after 4 weeks, the therapy was terminated.

### Evaluation of Outcome and Statistical Analysis

Data on effectiveness and symptoms were descriptive and based on assessment of clinical factors as well as a specialized questionnaire. Although subjective descriptions of symptoms were categorized in superordinate variables, analysis of data remained challenging and limited. All statistical calculations were performed with R for Windows software, release 3.4.0 (The R Foundation for Statistical Computing, University of Auckland, NZ, 2017). Clinical factors in correlation with effectiveness of the therapy were analyzed with Fisher's exact test for small group sizes. Results were considered significant at *p* < 0.05.

## Results

Of the 23 patients entered into the study, completed questionnaires were available in 17. Mean age of these 17 patients was 6.5 years (range 1–11 years). In 6 patients non-invasive sacral nerve stimulation was applied, but further participation in the study was withdrawn because of unwillingness to adhere to the study protocol, as well as due to lack of acceptance of the therapy by younger children. Treatment had never been terminated by physicians.

Main clinical characteristics are shown in Table 1. Gender was evenly distributed (9 boys, 8 girls). In 10/17 cases, defecation disorders had existed since birth or the first year of living. Mean age at beginning of symptoms was 2 years (range 0–4 years) and treatment of defecation disorders had been conducted for 5 years (mean value, range 1–10). Fecal incontinence was seen in 6 patients, soiling solely in further 5 patients. Defecation consistency was described in 15/17 patients as predominantly hard. Normal consistency could be achieved under conservative treatment in 2 cases only. Intermittent diarrhea, as known in overflow incontinence, occurred in 8 patients. Whereas, reduced eating habits could be seen in the majority of the cases, nausea and vomiting occurred seldom. Abdominal pain and psychological distress were seen in all patients (17/17). Two patients suffered from concomitant urinary incontinence and considered this the leading disorder. Exclusion of other causes of constipation, such as hypothyroidism or celiac disease, had been routinely conducted. All patients presented with chronic constipation refractory to conservative treatment. Treatment included lifestyle changes and improved toilet training in all cases. Further conservative options, such as biofeedback-training or regular disimpactations, had been used occasionally (biofeedback 7 patients of 6 patients with encopresis and one in trail application because of soiling, routine disimpactations 4/17 patients). In all patients, either single or combinatory therapy of oral and rectal medication (polyethylenglycol, klysmata) had been used previously within therapeutic management to ensure regular defecation. At the time of start of non-invasive sacral nerve stimulation, oral or rectal medication was necessary in 13/17 patients (see Table 1). In 4 patients, defecation was conducted after rectal manipulations at study entry (with or without sodium chlorid enemas), as medical therapy had been stopped previously due to side effects. All treatments (oral/rectal medication, rectal manipulations) were not altered without prior consultation. Prokinetics were not used within our study population at start of non-invasive sacral nerve stimulation. Their application was tested in 3 cases without sufficient success. Botox injections were applied in 3 patients of functional constipation and did not show a sufficiently lasting effect. Rectal biopsies for exclusion of Hirschsprung's disease were conducted in 16/17 patients. In 5 patients, Hirschsprung's disease had been histologically confirmed. Five patients had undergone prior abdominal surgery. In 4 cases, resection of aganglionosis due to Hirschsprung's diseases was conducted and in one patient laparoscopic appendectomy due to appendicitis.

**Table 1:** Patients' clinical characteristic.

	***n* = 17**
Sex	
Male	9
Female	8
Age (years, range)	1–11
Diagnosis	
Chronic constipation of idiopathic genesis	12
Hirschsprung's disease	5
Other conducted therapies	
None	9
Intramuscular Botox injections	3
Intestinal surgeries	5
Previous intestinal surgeries	
(Partial) rectal resection	4
Appendectomy	1
Medical treatment at the beginning of stimulation	
None	4
Polyethylenglycol *single medication* (Movicol junior®, Macrogol®, Dulcolax®)	7
Klysmata *single medication* (Microlax®, Microlist®)	2
Polyethylenglycol (Movicol junior®, Macrogol ®, Dulcolax®) *and* Klysmata (Microlax®, Microlist®)	4

Treatment characteristics, response, and complications are shown in Table 2. Neuromodulatory therapy was continued for a mean of 12.7 weeks (range 2–52 weeks). The duration of stimulation was conducted for a mean of 11 h per day (range 0.5–24 h). Stimulation intensity ranged from 3 to 9 V (mean 6 V). In 6 cases, electrodes had to be switched to twisted position as mentioned above, as routine settings did not lead to any therapeutic effect. We observed an improved therapeutic result in cases, which applied electric stimulation during night and for a prolonged time. Individual results are confirming this trend.

**Table 2:** Treatment characteristics of non-invasive sacral nerve stimulation.

	**Responders**	**Non-responders**	
	***n*** **= 10**	***n*** **= 7**	***P*****-values**
Duration of stimulation (weeks)			0.88
<4	1	2	
5–10	3	2	
11–20	4	1	
>21	1	1	
Not known	1	1	
Stimulation (time of day)			0.77
24 h	3	1	
Day	1	2	
Night	3	2	
Not known	3	2	
Duration of stimulation (hours per day)			0.91
24	3	1	
9–16	3	3	
8–4	1	1	
<3	2	2	
Not known	1	0	
Intensity of stimulation (V)			0.30
1–5	3	4	
6–10	6	2	
Not known	1	1	
Side effects: micturition			0.49
Yes	2	0	
No	7	6	
Not known	1	1	
Complications (skin irritation after application of electrodes)			0.09
Yes	4	0	
No	5	7	
Not known	1	0	

A change of symptoms was reported in 12 cases, with 10 (10/12) considering the increased regularity of stool frequency and decreased abdominal pain beneficial and satisfying. The clinical effect was considered to be low in 2 cases. In further 2 patients, a change in daily behavior was observed, leading to a substantial improvement in quality of life. In only 3 patients no clinical effect was observable (nor was any side effect). Individual improvements are presented in Table 3. In all cases considering the effectiveness of treatment satisfying (*n* = 10), all three primary outcome values (defecation frequency, abdominal pain, soiling) improved simultaneously. Symptom improvement was sustained in 5 of 12 patients reporting a change of symptoms. Although individual cases confirmed successful treatment, no significant correlation between therapeutic effect of non-invasive sacral nerve stimulation and clinical parameter was found.

**Table 3:** Outcome values of non-invasive sacral nerve stimulation, presenting the individual effectiveness in the included patients.

	***n* = 17**
Defecation frequency	
Improvement to daily/every second day	10
Improvement to increased frequency	2
No influence	5
Stool consistency	
Improvement to normal consistency	4
Changes in stool consistency	6
No influence	7
Abdominal pain	
Reduction of abdominal pain	13
No influence	4
Increase of abdominal pain	0
Soiling (affected cases: 11 patients)	
Improvement of soiling	9
No influence	2

*Overall effectiveness of the non-invasive sacral nerve stimulation therapy was seen in 59% of patients (n = 10, responders)*.

Positive side effects of therapy included increased agility during the day. Sleep disturbance did not occur. Parents described an essential improvement in the children's quality of life. Individual improvement of patients' well-being was not clearly related to objective measurements such as bowel movements, even though parents reported a more regular bowel pattern.

Complications of the therapy were rarely seen: urinary frequency increased in the 2 patients with previously diagnosed micturition disturbances. Frequent electrode detachment was seen in one patient and abdominal and dorsal skin irritation in 3, treated successfully with hypoallergenic electrodes.

## Discussion

Non-invasive sacral nerve stimulation is an innovative approach for treatment of chronic constipation in children, which was tested within a small heterogeneous group of patients in our institution. This external technique creates an electrical field that affects a large target area of the lower abdomen and pelvis and may thus have an advantage compared to direct nerve stimulation.

The mechanism of action can only be hypothesized based on knowledge of the effects of current on conductive tissue. A multimodal effect on sacral somatomotor, somatosensory, and autonomic nerves, as well as on the enteric nervous system can be postulated and may include influence on neuroplasticity of sensory nerve fibers in the skin, sensory and motor nerves in the spinal nerves, sympathetic and parasympathetic nerves, as well as direct activation and/or structural changes of cells of the enteric nervous system or interstitial cells of Cajal (intestinal pacemaker cells), and intestinal muscle cells (26). Additionally, it was suggested that there may be a change and strengthening in pelvic floor muscles, and a neuromodulation of the sacral reflexes as well (26, 27). Accordingly, we also assume that a modulation of central nervous functions via the brain-gut axis may occur (28). A lingering effect consists additionally of hormonal changes (29, 30).

The precise understanding of the impact of this stimulation on anatomical structures, and on endocrine, or other biochemical activities requires further research. However, there is an ongoing discussion and presentation of prokinetic effects of intestinal electric stimulation within experimental canine research (31, 32), failing to explain the exact pathophysiological mechanisms.

Although comparable approaches of electrostimulation in pediatric constipation are sparse, there are previously published neurostimulatory methods, presenting the application of interferential electric stimulation of the gut via external, adhesive surface electrodes in children and adolescents. An Australian research group focused within this context on the therapy of slow transit constipation (18–20, 33, 34). This transabdominal electrical stimulation approach (TES) was conducted with a medium beat frequency (80–150 Hz) of two out of phase channels producing a beating current and a pulse of 250 μs. Intensity levels could be chosen individually, aiming at a maximum current intensity below the pain threshold. Four electrodes were used to create a high field stability and a deep penetrating, interferential current (35). Commonly, electrical stimulation was applied physiotherapist-administered for 20 min in three times per week (20, 25). Within randomized case-control trails, Leong et al. was able to prove increased defecation frequency, improved stool consistency and decreased abdominal pain in 73% of the study population with lasting effects over more than 2 years in one third of the children (20). Accordingly, Yang et al. confirmed similar results in adults in 2014 (25). In more recent single cohort studies in children, Yik et al. (21) and Gunawan et al. (36) extended the application time within consisting variables to 1 h per day over 4–6 months, presenting equally significant results on the effectiveness of this new therapy. In 2016, Yik et al. (37) introduced the electrical stimulation therapy to a small group of children with chronic constipation due to anorectal retention, excluding patients with slow transit constipation or any underlying diseases. These patients' characteristics are most likely comparable to our heterogeneous cohort. However, this interferential stimulation was now applied with high-frequency pulses between 4080 and 4160 Hz daily for 1 h per day. Subsequent to these early studies, an application of stimulation was pursued in an autonomous use at home (19, 38, 39).

There is only one study so far, evaluating the effect of interferential electrical stimulation on patients with Hirschsprung's disease after resection of affected colic and rectal segments. Ladi-Seyedian et al. (22) was able to present results of a randomized case-control trail, adopting electrical variables and setting of the TES-approach (20 min of stimulation two times per week; field with 4 electrodes; individual intensity level 0–50 mA). However, the stimulation was applied with a low frequency of 5–25 Hz. Constipation symptoms and frequency of defecation per week were improved significantly in study group of interferential electrical stimulation in combination with behavioral therapy in comparison to their counterparts (only behavioral therapy).

Conclusively, positive influence of interferential electrical stimulation (short-term, medium- to high frequency) on any kind of chronic constipation could be seen (23). Nevertheless, the optimal stimulation parameters have not yet been determined and, while presented results in children are encouraging, multicenter studies are needed to confirm the positive impact on constipation and fecal incontinence (26).

Additionally, sacral nerve stimulation is a therapeutic approach for patients with chronic constipation and fecal incontinence and is well-established to prevent further intestinal surgeries. It is delivered via a percutaneous transforaminal surgery, placing electrodes for continuous and direct stimulation of the sacral nerve roots. This leads to reduction of fecal and urinary incontinence, improvement of stool frequency and consistency in chronic constipation, and a general improvement in quality of life with promising results (12, 40). However, the exact mechanism of action remains elusive and the discussion on outcomes, especially in children and adolescents, remains to be controversial (15, 16, 41).

We built on this knowledge and adapted this external electrical stimulatory method to our complex patients, referring to principles of sacral nerve stimulation. Our aim was therefore to apply a non-invasive and cost-efficient therapy for administration at home, with low frequency stimuli (15 Hz) in rectangular pulses of a single channel stimulation during a maximum of time to simulate physiological bowel movement. The basic calibration of the non-invasive sacral nerve stimulation was set before the initial appointments and could not be changed by patients. Standard parameters for SNS (13, 42) and data from Edel (43) for electro-medicine were used. We preferred to accept a wide range of intensity in our study (3–9 V), allowing to adapt to changing needs (e.g., post- irrigation) in the individual patient and promoting acceptance and compliance to the approach. An improved formation of the spanned field can be assumed in our cohort due to the reduction of electrodes to only one pair: with only one electrical field influencing the intestinal movement, efficacy and side effects are more controllable. In addition, the smaller number of adhesive electrodes carries less risk of skin irritation. Conclusively, these facts impede a direct comparison of both therapeutic approaches.

We are able to see an increased effect of electrical stimulation in application during nighttime, as it can additionally be seen in continuous application (only one out of 4 patients, applying continuous non-invasive stimulation over 24 h per day, was a non-responder to treatment). This might underline the physiological impact of the permanent stimulation and the increased motility during parasympathetic dominated phases.

Furthermore, we are able to present comparatively high efficacy as previously published. Efficacy was achieved in 12/17 patients and was considered strongly positive in the majority (10/12 patients). After discontinuation of stimulation, almost half of the patients confirmed sustained improvement (5 of the 12 patients reporting a change of symptoms). This effect of a neuromodulatory stimulation has already been reported (20). As previously discussed, we postulate that this carry-over effect may be related to the conditioning of the neurotransmitter system in children. Cross-linking mechanisms of the central nervous system have been described in biofeedback training in the context of therapy for urge incontinence in children (30).

Intestinal complications, such as previously reported diarrhea (18), were not observed. Intermittent electrode detachment and skin irritation led to improvement of fixation and the application of hypoallergenic electrodes in the four affected patients. When increased micturition frequency was reported (as it was in two patients with concomitant micturition disorders), it was resolved by change of position of the electrodes to adjust the electrical field.

This is a pilot study of non-invasive sacral nerve stimulation in a small and heterogeneous population of children and adolescents. Thus, there are certainly limitations to the presented results and conclusions may have to be drawn with caution. We are only able to confirm trends of efficacy. We established a protocol with clear therapeutic measurements, while responding to different needs of our patients. This results in variable stimulation times and intensities and certainly limits the evaluation and conclusion of our data, although this adaptability surely is one of the great strengths of the non-invasive treatment.

Furthermore, there is a lack of outcome measures. Although there are existing different standard daily defecation diaries and quality of life assessment forms, the heterogeneous clinical presentation of chronic constipation complicates the classification of symptoms and improvement. A substantial externalization remains thus to be a challenge and should be interpreted and categorized with great caution. We therefore focused primarily on an individual evaluation of defecation frequency, stool consistency, quality of life and abdominal pain. The reported observations will serve as a basis in the process for the development of new specialized questionnaires and research fields in subsequent studies. We are working on child-friendly questionnaires aiming to objectify results.

Finally, we are describing a therapeutic approach within a heterogeneous study population. Despite well-known diseases as Hirschsprung's, classification of chronic constipation in terms of idiopathic constipation or slow-transit constipation might still include a variety of different underlying causes. We therefore offered the treatment to all of the affected patients with chronic constipation, refractory to treatment. In our study cohort, 13/17 patients received medical therapy at study entry and all patients had received conservative treatment in accordance with a standardized treatment algorithm, but without sufficient therapeutic success (see Table 1).

Approximately one-fourth of patients presented with confirmed Hirschsprung's disease (*n* = 5). After multiple surgeries for resection of affected colic segments and regaining intestinal consistency, these patients are frequently still suffering from chronic constipation and remaining in difficult conditions. A new therapeutic option for this subgroup of patients with chronic constipation is essential for optimal treatment and improvement of quality of life. This small number of 5 patients within our study impedes to draw strong conclusions or subgroup analyses. Yet, non-invasive sacral nerve stimulation in these patients might have a therapeutic impact and specific effects on treatment changes have to be investigated further.

Conclusively, non-invasive sacral nerve stimulation might be a valuable option in pediatric constipation within a heterogeneous population and despite underlying diseases, as there is a great need to optimize treatment. The obtained results will have to be confirmed by an independent study population.

## Conclusion

Management options of chronic constipation in children and adolescents are limited, with options based mostly on best clinical practice without clear evidence. Thus, the demand for specialized research and innovative treatment pathways is urgent (44). With electrical stimulation via non-invasive sacral nerve stimulation, we propose a feasible method shown to be effective in this pilot study, which may add a valuable, complementary option to the treatment of this multicausal symptom complex. Main advantages, such as rapid therapeutic responses, high comfort and usability, minimal amount of side effects, and especially the individual management concerning treatment intensity and treatment time, should be noted. Strong conclusions though may be made cautiously based on our results of a small and heterogeneous study population, but further investigations on this topic seem to be crucial in clinical research of pediatric chronic defecation disorders. Our institution's algorithm for chronic constipation in childhood and adolescence assumes close collaboration with Pediatric Gastroenterology to find optimal treatment for these complex cases.

## Data Availability Statement

The datasets generated for this study are available on request to the corresponding author.

## Ethics Statement

The studies involving human participants were reviewed and approved by Ethics committee of Friedrich-Alexander University of Erlangen-Nürnberg, Erlangen, Germany. Written informed consent to participate in this study was provided by the participants' legal guardian/next of kin. Written informed consent was obtained from the minor(s)' legal guardian/next of kin for the publication of any potentially identifiable images or data included in this article.

## Author Contributions

SD, MB, VS, and RC: the concept of the study was designed and adjusted in progress. MK and SD: patients' data were acquired and included in a database. SD and VS: statistical analysis and interpretation of data was conducted. SD, MB, VS, and RC: the manuscript was written and optimized. All the authors contributed to the realization of the study and its publication.

### Conflict of Interest

The authors declare that the research was conducted in the absence of any commercial or financial relationships that could be construed as a potential conflict of interest.

## Supplementary Material

Supplementary File 1 Non-invasive sacral nerve stimulation - evaluation form” provides basis for evaluation of clinical parameters of patients under therapy with non-invasive sacral nerve stimulation.

## Abbreviations

SNS: sacral nerve stimulation
STC: slow-transit constipation
TES: transabdominal electrical stimulation.

## References

[1] Gupta S Schaffer G Saps M. Pediatric irritable bowel syndrome and other functional abdominal pain disorders: an update of non-pharmacological treatments. Expert Rev Gastroenterol Hepatol. (2018) 12:447–56. 10.1080/17474124.2018.1462699 29633902

[2] Adachi Y Ishii Y Yoshimoto M Yoshida Y Endo T Yamamoto H. Phenotypic alteration of interstitial cells of Cajal in idiopathic sigmoid megacolon. J Gastroenterol. (2008) 43:626–31. 10.1007/s00535-008-2207-4 18709485

[3] Sharma A Rao S. Constipation: pathophysiology and current therapeutic approaches. Hanb Exp Pharmacol. (2017) 239:59–74. 10.1007/164_2016_111 28185025

[4] Belsey J Greenfield S Candy D Geraint M. Systematic review: impact of constipation on quality of life in adults and children. Aliment Pharmacol Ther. (2010) 31:938–49. 10.1111/j.1365-2036.2010.04273.x 20180788

[5] Collins L Collis B Trajanovska M Khanal R Hutson JM Teague WJ. Quality of life outcomes in children with Hirschsprung disease. J Pediatr Surg. (2017) 52:2006–10. 10.1016/j.jpedsurg.2017.08.043 28927976

[6] Lee V Guthrie E Robinson A Kennedy A Tomenson B Rogers A. Functional bowel disorders in primary care: factors associated with health-related quality of life and doctor consultation. J Psychosom Res. (2008) 64:129–38. 10.1016/j.jpsychores.2007.09.004 18222126

[7] Stephens JR Steiner MJ DeJong N Rodean J Hall M Richardson T. Constipation-related health care utilization in children before and after hospitalization for constipation. Clin Pediatr. (2018) 57:40–5. 10.1177/0009922817691818 28627286

[8] Rao SS Rattanakovit K Patcharatrakul T. Diagnosis and management of chronic constipation in adults. Nat Rev Gastroenterol Hepatol. (2016) 13:295–305. 10.1038/nrgastro.2016.53 27033126

[9] Cottone C Tosetti C Disclafani G Ubaldi E Cogliandro R Stanghellini V. Clinical features of constipation in general practice in Italy. United European Gastroenterol J. (2014) 2:232–8. 10.1177/2050640614527283 25360307 PMC4212453

[10] Drossman DA. The functional gastrointestinal disorders and the Rome III process. Gastroenterology. (2006) 130:1377–90. 10.1053/j.gastro.2006.03.008 16678553

[11] Tabbers MM DiLorenzo C Berger MY Faure C Langendam MW Nurko S. Evaluation and treatment of functional constipation in infants and children: evidence-based recommendations from ESPGHAN and NASPGHAN. J Pediatr Gastroenterol Nutr. (2014) 58:258–74. 10.1097/MPG.0000000000000266 24345831

[12] Dinning PG Fuentealba SE Kennedy ML Lubowski DZ Cook IJ. Sacral nerve stimulation induces pan-colonic propagating pressure waves and increases defecation frequency in patients with slow-transit constipation. Colorectal Dis. (2007) 9:123–32. 10.1111/j.1463-1318.2006.01096.x 17223936

[13] Maeda Y O'Connell PR Lehur PA Matzel KE Laurberg S. Sacral nerve stimulation for faecal incontinence and constipation: a European consensus statement. Colorectal Dis. (2015) 17:O74–87. 10.1111/codi.12905 25603960

[14] Lu PL Asti L Lodwick DL Nacion KM Deans KJ Minneci PC. Sacral nerve stimulation allows for decreased antegrade continence enema use in children with severe constipation. J Pediatr Surg. (2017) 52:558–62. 10.1016/j.jpedsurg.2016.11.003 27887683

[15] Sulkowski JP Nacion KM Deans KJ Minneci PC Levitt MA Mousa HM. Sacral nerve stimulation: a promising therapy for fecal and urinary incontinence and constipation in children. J Pediatr Surg. (2015) 50:1644–7. 10.1016/j.jpedsurg.2015.03.043 25858097

[16] van der Wilt AA van Wunnik BP Sturkenboom R Han-Geurts IJ Melenhorst J Benninga MA. Sacral neuromodulation in children and adolescents with chronic constipation refractory to conservative treatment. Int J Colorectal Dis. (2016) 31:1459–66. 10.1007/s00384-016-2604-8 27294660 PMC4947479

[17] Vriesman MH Koppen IJN Camilleri M Di Lorenzo C Benninga MA. Management of functional constipation in children and adults. Nat Rev Gastroenterol Hepatol. (2020) 17:21–39. 10.1038/s41575-019-0222-y 31690829

[18] Chase J Robertson VJ Southwell B Hutson J Gibb S. Pilot study using transcutaneous electrical stimulation (interferential current) to treat chronic treatment-resistant constipation and soiling in children. J Gastroenterol Hepatol. (2005) 20:1054–61. 10.1111/j.1440-1746.2005.03863.x 15955214

[19] Ismail KA Chase J Gibb S Clarke M Catto-Smith AG Robertson VJ. Daily transabdominal electrical stimulation at home increased defecation in children with slow-transit constipation: a pilot study. J Pediatr Surg. (2009) 44:2388–92. 10.1016/j.jpedsurg.2009.07.063 20006033

[20] Leong LC Yik YI Catto-Smith AG Robertson VJ Hutson JM Southwell BR. Long-term effects of transabdominal electrical stimulation in treating children with slow-transit constipation. J Pediatr Surg. (2011) 46:2309–12. 10.1016/j.jpedsurg.2011.09.022 22152871

[21] Yik YI Clarke MC Catto-Smith AG Robertson VJ Sutcliffe JR Chase JW. Slow-transit constipation with concurrent upper gastrointestinal dysmotility and its response to transcutaneous electrical stimulation. Pediatr Surg Int. (2011) 27:705–11. 10.1007/s00383-011-2872-x 21373802

[22] Ladi-Seyedian SS Sharifi-Rad L Manouchehri N Ashjaei B. A comparative study of transcutaneous interferential electrical stimulation plus behavioral therapy and behavioral therapy alone on constipation in postoperative Hirschsprung disease children. J Pediatr Surg. (2017) 52:177–83. 10.1016/j.jpedsurg.2016.07.007 27524737

[23] Iacona R Ramage L Malakounides G. Current state of neuromodulation for constipation and fecal incontinence in children: a systematic review. Eur J Pediatr Surg. (2019) 29:495–503. 10.1055/s-0038-1677485 30650450

[24] Singh H Uhlmann J Khatun M Connor F Lumbosacral transcutaneous electrical stimulation in children with slow transit constipation: a pilot case series. J Hepatol Gastroenterol. (2017) 2:1–5. 10.14312/2399-8199.2017-1

[25] Yang Y Yim J Choi W Lee S. Improving slow-transit constipation with transcutaneous electrical stimulation in women: a randomized, comparative study. Women Health. (2017) 57:494–507. 10.1080/03630242.2016.1176098 27067259

[26] Moore JS Gibson PR Burgell RE. Neuromodulation via interferential electrical stimulation as a novel therapy in gastrointestinal motility disorders. J Neurogastroenterol Motil. (2018) 24:19–29. 10.5056/jnm17071 29291605 PMC5753900

[27] Kajbafzadeh AM Sharifi-Rad L Nejat F Kajbafzadeh M Talaei HR. Transcutaneous interferential electrical stimulation for management of neurogenic bowel dysfunction in children with myelomeningocele. Int J Colorectal Dis. (2012) 27:453–8. 10.1007/s00384-011-1328-z 22065105

[28] Bittorf B Ringler R Forster C Hohenberger W Matzel KE. Cerebral representation of the anorectum using functional magnetic resonance imaging. Br J Surg. (2006) 93:1251–7. 10.1002/bjs.5421 16758465

[29] Fuentes CJ Armijo-Olivo S Magee DJ Gross DP A preliminary investigation into the effects of active interferential current therapy and placebo on pressure pain sensitivity: a random crossover placebo controlled study. Physiotherapy. (2011) 97:291–301. 10.1016/j.physio.2011.01.001 22051585

[30] Zhu MH Sung IK Zheng H Sung TS Britton FC O'Driscoll K. Muscarinic activation of Ca2+-activated Cl- current in interstitial cells of Cajal. J Physiol. (2011) 589(Pt 18):4565–82. 10.1113/jphysiol.2011.211094 21768263 PMC3208225

[31] Chen S Liu L Guo X Yao S Li Y Chen S. Effects of colonic electrical stimulation using different individual parameter patterns and stimulation sites on gastrointestinal transit time, defecation, and food intake. Int J Colorectal Dis. (2016) 31:429–37. 10.1007/s00384-015-2457-6 26607906

[32] Wang WF Yin JY De Dz Chen J. Acceleration of small bowel transit in a canine hypermotility model with intestinal electrical stimulation. J Dig Dis. (2015) 16:135–42. 10.1111/1751-2980.12220 25495658

[33] Clarke MC Catto-Smith AG King SK Dinning PG Cook IJ Chase JW. Transabdominal electrical stimulation increases colonic propagating pressure waves in paediatric slow transit constipation. J Pediatr Surg. (2012) 47:2279–84. 10.1016/j.jpedsurg.2012.09.021 23217889

[34] Clarke MC Chase JW Gibb S Hutson JM Southwell BR. Improvement of quality of life in children with slow transit constipation after treatment with transcutaneous electrical stimulation. J Pediatr Surg. (2009) 44:1268–72; discussion 72. 10.1016/j.jpedsurg.2009.02.031 19524752

[35] Palmer ST Martin DJ Steedman WM Ravey J. Alteration of interferential current and transcutaneous electrical nerve stimulation frequency: effects on nerve excitation. Arch Phys Med Rehabil. (1999) 80:1065–71. 10.1016/S0003-9993(99)90062-X 10489010

[36] Gunawan RHJ Southwell BR Non-invasive medical device for the treatment of chronic constipation: 1. Proof-of-principle study in children. Abstracts in Motility/Neurogastroenterology. J Gastroenterol Hepatol. (2017) 32 (Suppl. 2):172–8. 10.1111/jgh.13897

[37] Yik YI Stathopoulos L Hutson JM Southwell BR. Home transcutaneous electrical stimulation therapy to treat children with anorectal retention: a pilot study. Neuromodulation. (2016) 19:515–21. 10.1111/ner.12451 27293084

[38] Yik YI Hutson J Southwell B. Home-based transabdominal interferential electrical stimulation for six months improves paediatric slow transit constipation (STC). Neuromodulation. (2018) 21:676–81. 10.1111/ner.12734 29164818

[39] Yik YI Ismail KA Hutson JM Southwell BR. Home transcutaneous electrical stimulation to treat children with slow-transit constipation. J Pediatr Surg. (2012) 47:1285–90. 10.1016/j.jpedsurg.2012.03.037 22703807

[40] Carrington EV Evers J Grossi U Dinning PG Scott SM O'Connell PR. A systematic review of sacral nerve stimulation mechanisms in the treatment of fecal incontinence and constipation. Neurogastroenterol Motil. (2014) 26:1222–37. 10.1111/nmo.12388 25167953

[41] Dewberry L Trecartin A Pena A Pierre MS Bischoff A. Systematic review: sacral nerve stimulation in the treatment of constipation and fecal incontinence in children with emphasis in anorectal malformation. Pediatr Surg Int. (2019) 35:1009–12. 10.1007/s00383-019-04515-z 31256299

[42] Matzel KE Chartier-Kastler E Knowles CH Lehur PA Munoz-Duyos A Ratto C. Sacral neuromodulation: standardized electrode placement technique. Neuromodulation. (2017) 20:816–24. 10.1111/ner.12695 28975677

[43] Edel H Fibel der Elektrodiagnostik und Elektrotherapie, 6. Berlin: Auflage; Verlag Gesundheit GmbH (1991).

[44] Siminas S Losty PD. Current surgical management of pediatric idiopathic constipation: a systematic review of published studies. Ann Surg. (2015) 262:925–33. 10.1097/SLA.0000000000001191 25775070

