# Supplementary material for: A Pilot Study of Non-invasive Sacral Nerve Stimulation in Treatment of Constipation in Childhood and Adolescence
Source: Front Pediatr. 2020 Apr 16;8:169. doi: 10.3389/fped.2020.00169 (PMC7176809; doi:10.3389/fped.2020.00169)
Supplement: Supplementary File 1 — Non-invasive sacral nerve stimulation - evaluation form” provides basis for evaluation of clinical parameters of patients under therapy with non-invasive sacral nerve stimulation. [file Data_Sheet_1.PDF]

Department of Pediatric Surgery
Non-invasive sacral nerve stimulation
Evaluation form

Current symptoms

Defecation frequency	O > 1-1x/day	O every 2-3. day	O < 2x/week
Stool consistency	O hard	O soft	O fluid
Abdominal pain	O yes	O no	
Defecation pain	O yes	O no	
Flatulence	O yes	O no	
Fecal incontinence	O yes	O no	
Soiling	O yes	O no	
Enuresis	O yes	O no	

Please specify symptom characteristics: frequency? dependence? further symptoms?

Current therapy

Drugs

O None
O Polyethylenglycol single medication (Movicol junior®, Macrogol®, Dulcolax®)
O Klysmata single medication (Microlax®, Microlist®)
O Prokinetics
O Others

Please specify dose and compliance

Other therapies

O Rectal manipulations
O Rectal enema (sodium chloride)
O Biofeedback
O Lifestyle changes
O Other, please specify

Non-invasive sacral nerve stimulation

Date of beginning ____________________

Position of electrodes	○ Back left	○ Back right	Change of position?	Date ____________
Intensity of stimulation (0-10V)?				
Stimulation time per day?	○ 1- 3 hours	○ 3-8 hours	○ 8-12 hours	○ 12-24 hours
Time of stimulation?	○ daytime	○ nighttime	○ whole day	○ variable
Complications due to stimulation?	○ no	○ yes		
Notes: interruption of therapy? problems? changes in effects? effects on further symptoms? complications?				

Quality of life under therapy

Is the therapy considered to be successful?	○ no	○ yes	○ unclear
Is there an improvement of attitude?	○ no	○ yes	○ unclear
Is there an improvement of activity/liveliness?	○ no	○ yes	○ unclear

Quality of life of the patient?	○ 1 poor	○ 2	○ 3	○ 4	○ 5	○ 6	○ 7 excellent
Further notes on quality of life of the patient?							
